# Shanghai cognitive intervention of mild cognitive impairment for delaying progress with longitudinal evaluation-a prospective, randomized controlled study (SIMPLE): rationale, design, and methodology

**DOI:** 10.1186/s12883-018-1100-x

**Published:** 2018-07-24

**Authors:** Yiqi Lin, Binyin Li, Huidong Tang, Qun Xu, Yuncheng Wu, Qi Cheng, Chunbo Li, Shifu Xiao, Lu Shen, Weiguo Tang, Hui Yu, Naying He, Huawei Lin, Fuhua Yan, Wenwei Cao, Shilin Yang, Ye Liu, Wei Zhao, Dong Lu, Bin Jiao, Xuewen Xiao, Lin Zhou, Shengdi Chen

**Affiliations:** Department of Neurology & Institute of Neurology, Rui Jin Hospital affiliated to Shanghai Jiao Tong University School of Medicine, Shanghai, 200025 China

**Keywords:** Mild cognitive impairment, Cognitive training, China, Longitude evaluation, Randomized controlled trial

## Abstract

**Background:**

Mild cognitive impairment is an early stage of Alzheimer’s disease. Increasing evidence has indicated that cognitive training could improve cognitive abilities of MCI patients in multiple cognitive domains, making it a promising therapeutic approach for MCI. However, the effect of long-time training has not been widely explored. It is also necessary to evaluate the extent how it could reduce the convertion rate from MCI to AD.

**Methods/design:**

The SIMPLE study is a multicenter, randomized, single-blind prospective clinical trial assessing the effects of computerized cognitive training on different cognitive domains in MCI patients. It is carried out in 7 centers in China. The study population includes patients aged 50–85, and they are randomly allocated to the training or control group. The primary outcome is to compare the conversion rate of MCI within 36-month follow-up. Structural and functional MRI will be used to interpret the effect of cognitive training. The cognitive training comprises a variety of games related with cognitive domains such as attention, memory, visualspatial ability and executive function. We cautiously set 50% reduction in the rate of conversion as estimated effect. With 80–90% statistical power and 12% as the overall probability of conversion within the study period, 600–800 patients are finally required in the study. The first patent has been recruited in April 2017.

**Discussion:**

Previous studies suggested the benefit of cognitive training for MCI, but neither long-time nor Chinese culture were investigated. The SIMPLE designs and utilizes an improved computerized cognitive training approach and assesses its effects on MCI progress. In addition, neural activities explaining the effects on cognition function changes will be revealed, which could in turn to imply more useful therapeutic approaches.

**Trial registration:**

ClinicalTrials.gov Identifier: NCT03119051.

**Electronic supplementary material:**

The online version of this article (10.1186/s12883-018-1100-x) contains supplementary material, which is available to authorized users.

## Background

Alzheimer’s disease (AD) is one of the most common neurodegenerative diseases. Its symptoms usually emerge gradually after the age of 65 and get worse over time. The disease seriously impaired cognitive functions and daily living abilities, posing heavy care and economic burden on patients’ families and the society. The American Cognitive Function and Aging Study (ACFAS) indicates that the prevalence of AD among people over 65 is 6.5% [1]. In Shanghai, one of the most biggest cities in China, the prevalence of cognition impairment in people over 60 in rural regions and over 55 in urban areas are reported to be 7 and 8.38% respectively [2, 3]. The number of AD patients in China is estimated to reach 23.3 million in 2030 [4].

So far there have been a few effective treatment for AD, though cognitive decline may be slowed down by medications like cholinesterase inhibitors and N-methyl-D-aspartate receptor antagonists. Once dementia happens, no treatment could stop or reverse disease progression by now. The latest studies of AD medications, for example, phase II or III clinical trials using monoclonal antibodies for immunomodulatory therapy [5, 6], all failed in the end. In April of 2011, the National Institute on Aging Alzheimer’s Association (NIA-AA) extended the definition of AD and described three stages of AD [7]. The updated diagnostic guidelines describe preclinical stage before dementia: asymptomatic stage with underlying amyloid deposit and neural injury. In the mildly symptomatic predementia stage, mild cognitive impairment due to AD (MCI) is marked by mild symptoms (no interference with independence) with at least one positive biomarker, including β-amyloid deposits or evidence of neuronal injury. Among the three stages in the NIA-AA guideline, MCI is of greatest significance for clinical interventions. First, approximately 10–12% of MCI patients progressed to AD every year and the 3-year MCI-AD conversion rate reached up to 25–30%. Overall, more than 60% of MCI patients developed AD [8]. So interventions for MCI patients are highly necessary. Secondly, presence of cognitive symptoms makes it possible for identification and diagnosis. Finally, since symptoms are relatively mild, patients could cooperate well with various therapeutic interventions.

Pharmacological and non-pharmacological treatments have been greatly concerned about their effects. Donepezil, rivastigmine, galantamine, and memantine are the drugs presently approved by the Food and Drug Administration (FDA) for treatment of AD. However, both cholinesterase inhibitors (ChEIs) and antiglutamatergic treatments are not indicated for MCI patients. A review and meta-analysis concluded that treatment with ChEIs merely affected MCI progression to dementia or improved cognitive test scores [9]. In one study of data from Alzheimer’s Disease Neuroimaging Initiative, MCI patients who received ChEIs with or without memantine were more impaired, showed greater decline in scores, and progressed to dementia sooner than patients who did not receive ChEIs [10].

In addition, as cognitive symptoms of MCI patients are still mild, drug compliance could be another problem. Moreover, costs and side effects of drugs are important negative factors of early medical treatment.

In recent years, cognitive training has gradually become an important therapeutic approach due to its convenience and effectiveness. It describes a group of brain games exercising mental processing ability such as attention, memory, calculation and so on [11]. An increasing number of studies have proved that cognitive training has beneficial effects on delaying cognitive decline in the elderly. The Advanced Cognitive Training for Independent and Vital Elderly (ACTIVE) study which did a 5-year follow-up of 2802 healthy aging people found that there was a significantly smaller proportion of cognitive decline and higher score of living abilities in the intervention group receiving various cognitive training [12]. Similar effects of cognitive training are also found in MCI patients. Sylvie Belleville et al. [13] found that both MCI patients and healthy elders who received an 8-week intervention focusing on teaching episodic memory strategies had better performance in episodic memory assessment than the control group. An randomized clinical trial of 100 MCI patients found that 6-month cognitive training improved significantly cognitive functions of the intervention group in multiple domains and this improvement was well maintained during a 18-month follow-up [14].

However, there are some limitations in present cognitive training [15]. First, not all cognitive domains are involved in the training. For example, in the ACTIVE study [16], participants received training related with the ability of memory, attention and reasoning, while other cognitive domains like visuospatial and executive functions were not involved. Second, in the traditional pencil-and-paper training, difficulty levels hardly vary from person to person according to their different cognitive abilities. In addition, most clinical trials only performed neuropsychological assessment without objective biomarkers or imaging evaluation. Finally, most clinical trials lack a stable longer-term follow-up of a large number of MCI patients.

Computerized cognitive training has been considered as a convenient, flexible and scalable intervention, which might protect cognition from aging and AD [17]. A meta-analysis suggested its efficacy on global cognition, select cognitive domains, and psychosocial functioning in MCI patients [18]. Our preliminary data analysis suggested that computerized cognitive training improved the MCI participants’ cognitive functions. We once performed a human-computer interaction-based comprehensive training for 66 MCI or dementia patients in Shanghai, and found that the training delayed cognitive decline in visualspatial ability [19]. Thus, based on the previous studies, a 3-year follow-up prospective multi-center trial will be conducted for MCI patients to investigate the role of the computerized-cognitive training in cognitive improvement and its possible mechanisms. Neuropsychological assessment and both structural and functional neuroimaging will be used for evaluation at baseline and follow-up.

## Methods/design

### Study aims

The primary aim of SIMPLE is to evaluate the effect of the computerized cognitive training on the conversion rate of MCI to AD in the 3-year follow-up.

The secondary aims of this study are to investigate how the cognitive training affects global cognitive function, daily living function and brain activities, and to determine whether patients should receive such intervention at MCI stage.

The main research topics are:

1. Are there significantly less MCI patients convert to AD if they receive computerized cognitive training in the 3 years period?
2. Could the scores of cognitive assessments and daily living activities be improved after training?
3. Does computerized cognitive training have effect on the brain structure or activities (both regional and neural connectivity) changes? If so, how are they linked with cognitive function?

### Study design

SIMPLE is a 3-year multicenter prospective single-blind clinical cohort study with two parallel arms in 1:1 ratio. Patients are randomized into the training group the control group (absence of training). The total training dose will be around 200 h, with 3–4 sessions per week and 30 min per session. Though dose effect was not well studied, we will give the training dose that is acceptable by MCI patients according to previous studies and our preliminary data. Global cognitive function and daily living activity will be assessed at baseline and every 6 months afterwards (in month 6, 12, 18, 24, 30 and 36), while specific cognitive domains (memory, executive function, attention and language) will be evaluated at baseline and every 12 months afterwards (in month 12, 24 and 36). In addition, brain structure and function will be assessed by MRI at baseline and in month 12 and 36. During the follow-up period, medications for dementia (ChEIs or memantine) are not indicated. If a patients converts to AD during follow-up, pharmacological therapy starts. Cognitive assessments for all cognitive domains and MRI will be performed at that time. Meanwhile, telephone follow-ups will be conducted every two weeks in both groups. As an single-blind trial, neuropsychological assessor will be blinded to the group allocation. The total duration of the study will be approximately 5 years, from June 2017 (first in) until June 2022 (last out).

### Patients

In total, 600–800 MCI patients will be enrolled from memory clinic of four centers in Shanghai, one in Zhejiang Province, one center in Hu’nan Province and one in An’hui Province: Ruijin Hospital affiliated to Shanghai Jiaotong University School of Medicine, Renji Hospital affiliated to Shanghai Jiaotong University School of Medicine, Shanghai General Hospital affiliated to Shanghai Jiaotong University School of Medicine, Shanghai Mental Health Center, Suzhou municipal hospital in Anhui Province, Zhoushan Hospital in Zhejiang Province and Xiangya Hospital in Hu’nan Province. Among them, Ruijin Hospital is the leader of SIMPLE, in charge of patients enrollment, quality control and ethical problems. The inclusion and exclusion criteria are shown as follows.

### Inclusion criteria are

- ▪ Male or female, aged 50–85;
- ▪ MCI diagnosed according to the National Institute on Aging-Alzheimer’s Association (NIA-AA) workgroups [20];
- ▪ 24 ≤ Mini-Mental State Examination (MMSE) ≤28;
- ▪ The Hamilton Depression Scale/17-item (HAMD) score ≤ 10;
- ▪ Not on medication for dementia (Table 1);
- ▪ MRI T2 weighted imaging (T2WI) scan: if aged ≤70, Fazeca scale for White Matter lesions rating level ≤ 1; if > 70 years, white matter damage rating scale ≤2. The number of lacunar infarcts larger than 2 cm in diameter ≤ 2. No infarcts in hypothalamus, entorhinal cortex, para-hypothalamus, cortical and subcortical cavity infarction near gray matter nuclei. The medial temporal lobe atrophy (MTA) score: Grade II or above.
- ▪ Accompanied by reliable caregivers (at least intensive contact of 4 days every week and 2 h every day) who are expected to participate in the follow-up visits and provide useful information for scores of scales;
- ▪ Education level: primary school (grade 6) or above.
- ▪ Expected good compliance with the therapy and follow-up. Each patient gives a written informed consent, and the study has been approved by Ruijin Hospital’s ethical committees.

**Table 1:** Forbidden and restricted medication in the trial

Forbidden medication	Cholinesterase inhibitors
	N-methyl-D-aspartate (NMDA) antagonists
	Synthetic adrenal corticosteroids
	Central nervous system stimulants
	Chinese and western cognitive improvement medication
Restricted medication^a^	Antipsychotics
	Antidepressants
	Sedative-hypnotics^b^

Restricted medication^a^: The dose of these medications should not be increased during the trial. Patients with these medications for a long time should keep the dose stable

Sedative-hypnotics^b^: If necessary, temporary use of zopiclone, alprazolam or estazolam is allowed. Last dose before psychological assessment should not be given later than 22:00 last night

### Exclusion criteria are

- ▪ Cognitive decline caused by other diseases (including but not limited to: cerebrovascular disease, central nervous system infections, Parkinson’s disease, metabolic encephalopathy, deficiency of folic acid/vitamin B12 and hypothyroidism);
- ▪ Medical history of other neurological disorders (including stroke, Parkinson’s disease, epilepsy, etc.);
- ▪ Psychiatric disorders defined by Diagnostic and Statistical Manual of Mental Disorders (DSM)-IV criteria;
- ▪ Severe disease of heart, lung, liver, kidney or hematopoietic system;
- ▪ History of alcohol or drug abuse;
- ▪ Participation in other clinical trials within 30 days before the screening of this study;
- ▪ Other reasons that make one inability to complete the study.

### Withdrawal criteria are

Patients should withdraw from the trial if they meet the following criteria:

- ▪ Poor compliance to the protocol;
- ▪ A serious complication or other severe disease happens in the trial.
- ▪ Patients refuse to be followed up or become lost at the point of follow-up.

### Endpoint criteria

Patients finish their training when either of the criteria is met:

- ▪ Patients finish 3-year cognitive training;
- ▪ Patients meet the diagnostic criteria of dementia due to AD, according to the National Institute on Aging-Alzheimer’s Association (NIA-AA) workgroups [7].

### Recruitment, screening and run-in period

Patients will be recruited from the memory clinic of 7 centers above. During the first week, each patient will perform MMSE and HAMD as screen. Then eligible patients will enter a run-in period of 2 weeks, during which they will receive blood tests, electrocardiogram, cranial MRI and a basic computer operation test. The blood tests contain complete blood count, liver and kidney function test, syphilis, folic acid level, vitamin B12 level, thyroid function and ApoE genotype. The purpose of the run-in period is to collect basic clinical data, rule out other causes of cognitive decline and evaluate participants’ compliance. Then all the eligible patients will be registered before baseline assessment.

### Baseline assessment and randomization

Baseline demographics include sex, age, education level, occupation, concomitant disease and medications. Physical exercise and diet style will also be recorded.

A neuropsychological battery for multiple cognitive domains will be performed by neuropsychological assessors who are blind to the following grouping. These tests included MMSE, the Chinese version of Alzheimer’s Disease Assessment Scale-Cognitive subscale(ADAS-Cog) [21], Alzheimer’s Disease Cooperative Study-Activities of Daily Living (ADCS-ADL) [21], the Auditory Verbal Learning Test (AVLT)-Huashan version [22], the Shape Trail Test (STT, including Part A and B) [23], the Rey-Osterrieth Complex Figure Test (CFT) [24], the Stroop Color-World Test (SCWT) and Boston Naming Test (see Additional file 1).

After the baseline assessment, patients will be randomly allocated into two groups, which will be managed by Ruijin Hospital with interactive web response system. The stratification factors will be balanced between two groups when allocation, including education level (university or not) and age (older than 65 years or not). Assessors will be specific in each center and blinded to the grouping.

### Ethics and informed consent

Conformed to the ethical principles proclaimed in the Declaration of Helsinki in 1964, and revised in Tokyo in 2004, the study protocol has been reviewed and thus approved by independent ethics committees in the Ruijin Hospital, which is the leading center of the study. Before the inclusion, written informed consent forms will be signed by patients or their surrogate family member after a detailed explanation. The SIMPLE study is registered with Chinese Trial Registry.gov and ClinicalTrial.gov (Register number: ChiCTR-INR-16008959 and NCT03119051).

### Intervention and follow-up

All the participants in the intervention group will login an online training website with their own passwords by either cell phone or tablet computer. A variety of cognitive domains will be involved in the training including memory, calculation, attention, visualspatial and executive skills. The difficulty level of each game will be adjusted automatically according to patients’ last training results so that patients’ accuracy rate will be kept between 70 and 80%. Patients will be required to play 3–4 times of 30 min’ training game every week. Training time and results will be uploaded immediately to a web server which could be monitored by investigators. In contrast, participants in the control group will be required to neither alter their daily lifestyle nor receive any additional cognitive training.

**Fig. 1:**
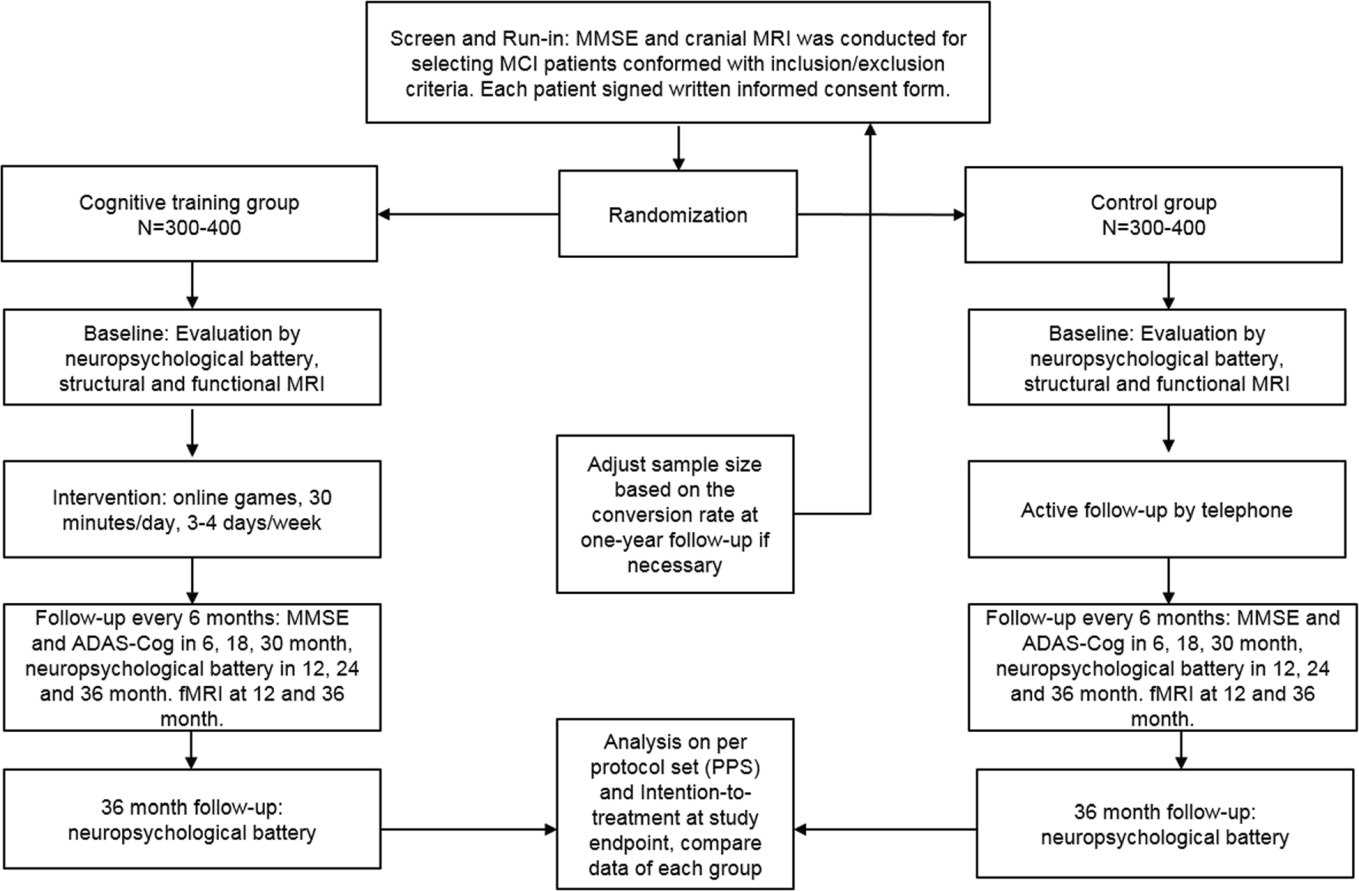
The SIMPLE study flowchart. The flowchart included the general protocol of recruitment, grouping, follow-up and final analysis

Investigators will conduct telephone follow-ups for both groups every two weeks to obtain their feedback and offer encouragement. On the other hand, follow-up assessment will be performed every six months (in month 6, 12, 18, 24, 30, 36 ± 2 weeks). In month 6, 18, 30, brief assessment including MMSE, ADAS-Cog and ADL would be done, while in month 12, 24 and 36, the neuropsychological battery at baseline for multiple cognitive domains will be performed. MRI is given in month 12 and 36 (Fig. 1).

### Outcome measures

The primary outcome is the incidence of AD during the follow-up. The diagnosis of AD will be made according to NIA-AA criteria. The performance in each cognitive domain will be compared as secondary outcomes. ADAS-Cog will be used to assess the global cognitive performance of MCI patients. Memory will be assessed using the AVLT-H. Attention and executive functions will be evaluated by TMT and the Stroop task. Language will be assessed by Boston naming test (30-item version). Visual spatial ability and visual memory will be evaluated by the Rey–Osterrieth complex figure test (CFT). Activities of daily living will be assessed by ADCS-ADL. Structural and functional MRI will help to uncover the potential neural activity changes during training. The schedule of all the outcome measures is shown in the Table 2.

**Table 2:** Outline and timelines of the trial

	Screening	Run-in	Baseline	1st Visits	2nd Visit	3rd Visit	4th Visit	5th Visit	Endpoint^a^
Time Points	−3 weeks	−2 weeks	0	6 months	2 months	18 months	24 months	30 months	36 months
Written informed consent		×							
Inclusion/exclusion criteria	×	×	×						
Blood test		×							
Electrocardiogram		×							
Cranial MRI examination		×			×				×
MMSE	×		×	×	×	×	×	×	×
ADAS-Cog			×	×	×	×	×	×	×
ADCS-ADL			×	×	×	×	×	×	×
HAMD/GDS	×		×		×		×		×
AVLT			×		×		×		×
TMT			×		×		×		×
CFT			×		×		×		×
Stroop Test			×		×		×		×
Boston Naming Test			×		×		×		×

^a^Endpoint is either the ending of 36-month follow-up or the diagnosis of AD during follow-up

### Neuroimaging markers

Structural and functional brain MRI will be performed at baseline, one-year follow-up and endpoint (Table 2). The MRI protocol is described in the Table 3. In structural MRI analysis, medial temporal lobe atrophy visual rating scale is used to indicate the level of atrophy in the medial temporal lobes (ranging from 0 to 3) [25]. According to cognitive ability changes, we also use voxel based morphometry (VBM) to quantitatively compare the volume of specific areas in brain. With regard to functional MRI, we use blood oxygen level dependent (BOLD) signal to find out functional changes related with cognitive performance changes. Fractional amplitude of low frequency fluctuation (fALFF), regional homogeneity (Reho) will be used to represent regional neural activities, while neural network changes are indicated by functional connectivity [26, 27].

**Table 3:** Brain MRI protocol

Sequence name/Acquisition method	Field of View (mm)	Matrix	Slices	Inter-slice gap (mm)	Thickness (mm)	Voxel (mm^3^)	TR /TE (ms)
3D FSPGR	256 × 256	256 × 256	192	0	1	1 × 1 × 1	5.52/1.72
BOLD	240 × 240	64 × 64	33	0.7	3.5	3.5 × 3.5 × (3.5 + 0.7)	2000/30

*Abbreviations: TR* repetition time, *TE* echo time, 3D FSPGR three-dimensional fast spoiled gadient-recalled

### Sample size and statistical analysis

Sample size was estimated based on conversion rate from MCI to AD in the SAS study [28]. In the SAS sample, it was 6.0 per 100 person-years. In the three-year follow-up, the overall probability of conversion event would be 17%. We use hazard ratio (HR) to represent the converting difference of the training group per unit time as the control group. HR represents instantaneous risk over the all period, and is clinically significant due to its indication risks that happen before the endpoint. A power calculation was performed with the online freeware program by “time-to-event” method [29] (http://powerandsamplesize.com).

Due to HR as an almost unprecedented outcome in such sort of trial, we cautiously set 50% reduction in the rate of conversion as estimated effect. With 80–90% statistical power and 12% as the overall probability of conversion within the study period, 544–728 patients in total are considered the minimal number necessary to estimate the hazard ratio. Considering 10% dropout, we plan to enroll approximately 600–800 patients, with 300–400 patients in each group.

As an exploring study, we used per-protocol set instead of intention-to-treat (ITT) analysis as primary endpoint analysis in order to strictly estimate the effect of training. If participants were indicated cholinesterase inhibitors or memantine, or they could not insist on training, they would be excluded from per-protocol sets. ITT approach will also be done to avoid the effects of crossover and dropout. The results of these two analysis will be carefully interpreted.

The randomization is stratified by age and education years according to the Ruijin center using SAS statistical software (SAS Institute Inc., Cary, NC, USA) by a statistician (Qi Cheng) with no access to information on the patients or physicians. Patients will be randomized in a ratio of 1:1 to receive the training or only follow-up. In the randomization process, the randomized code is generated and assigned to physicians in a sealed envelope by the statistician. Afterwards, the sealed randomization codes are sent out to each center. The odd number means the training group, while the even number means the control group. If the patient cannot be trained because of difficulty in operating computer or cellphone, the patient will be managed as off-trial.

Continuous variables (scores of each cognitive assessment) will undergo mixed model analysis in the two groups for the secondary outcomes. At the same time, the effect of ApoE genotype on primary outcome will be observed through analysis of subgroups divided by ApoE genotype in both groups. With regard to MRI, VBM measures and BOLD signal will be used to find out structural and functional changes related with cognitive performance changes.

## Discussion

The SIMPLE trial is leaded by Ruijin Steering Committee (Binyin Li, Huidong Tang, Shengdi Chen), which is assisted by an independent data safety and monitoring board from Public Health College of Shanghai Jiaotong University School of Medicine (leaded by Qi Chen). The Board includes epidemiologists and statisticians, who are not otherwise participating in the trial. The Steering Committee members and principal investigators of each center (Qun Xu in Shanghai Renji Hospital, Yunchen Wu in Shanghai General Hospital, Chunbo Li and Shifu Xiao in Shanghai Mental Health Center, Hui Yu in Suzhou municipal hospital, Anhui Province, Weiguo Tang in Zhoushan Hospital, Zhejiang Province, Lu Shen in Xiangya Hospital, Hu’nan Province) are in charge of the ethics and patient safety. They will have a face-to-face or online meeting every month to monitor the status and progress of the trial. The diagnosis of AD will be reviewed by independent experts in each center and steering committee. The sponsor of the SIMPLE trial is the Clinical Research Center, Shanghai Jiao Tong University School of Medicine. The sponsor has no influence on the study design, data collection, data analysis, and final drafting of the manuscript.

The primary object of this multicenter randomized clinical trial is to explore the effect of multi-model online cognitive intervention for MCI patients. Seven centers are enrolled in the trial and the first patient will be recruited into the study in April 2017. The results are expected for publications in 2022.

## Additional file

Additional file 1: Description of neuropsychological battery. Here is the description of neuropsychological assessments used in in the SIMPLE study, including their versions, scores and clinical significance. (DOCX 35 kb)

## Abbreviations

ACFAS: the American Cognitive Function and Aging Study
ACTIVE: The Advanced Cognitive Training for Independent and Vital Elderly
AD: Alzheimer’s disease
ADAS-Cog: Alzheimer’s Disease Assessment Scale-Cognitive subscale
ADCS-ADL: Alzheimer’s Disease Cooperative Study-Activities of Daily Living
AVLT: Auditory Verbal Learning Test
BOLD: Blood oxygen level dependent
CFT: Complex Figure Test
ChEIs: cholinesterase inhibitors
DSM: Diagnostic and Statistical Manual of Mental Disorders
fALFF: Fractional amplitude of low frequency fluctuation
FDA: Food and Drug Administration
HAMD: Hamilton Depression Scale
HR: Hazard ratio
ITT: intention-to-treat
MCI: mild cognitive impairment due to AD
MMSE: Mini-Mental State Examination
NIA-AA: National Institute on Aging Alzheimer’s Association
Reho: Regional homogeneity
SCWT: Stroop Color-World Test
STT: Shape Trail Test
T2WI: T2 weighted imaging
VBM: Voxel based morphometry

## References

[1] Matthews FE (2013). A two-decade comparison of prevalence of dementia in individuals aged 65 years and older from three geographical areas of England: results of the cognitive function and ageing study I and II. Lancet.

[2] Zhang ZX (2005). Dementia subtypes in China: prevalence in Beijing, Xian, shanghai, and Chengdu. Arch Neurol.

[3] Yao YH, Xu RF, Tang HD, Jiang GX, Wang Y, Wang G (2010). *Cognitive Impairment and* Associated factors among the elderly in the shanghai suburb: findings from a low-education population. Neuroepidemiology.

[4] Xu J (2017). The economic burden of dementia in China, 1990-2030: implications for health policy. Bull World Health Organ.

[5] Salloway S, Sperling R, Brashear HR (2014). *Phase 3 trials of solanezumab and bapineuzumab for Alzheimer's disease*. N Engl J Med.

[6] Richard Dodel AR (2013). Peter Bartenstein et al, *Intravenous immunoglobulins for the treatment of mild to moderate Alzheimer’s disease: a phase II, randomised, doubleblind, placebo-controlled dose-finding trial*. Lancet Neurol.

[7] McKhann GM (2011). The diagnosis of dementia due to Alzheimer's disease: recommendations from the National Institute on Aging-Alzheimer's Association workgroups on diagnostic guidelines for Alzheimer's disease. Alzheimers Dement.

[8] Grundman M (2004). Mild cognitive impairment can be distinguished from Alzheimer disease and normal aging for clinical trials. Arch Neurol.

[9] Russ TC, Morling JR (2012). Cholinesterase inhibitors for mild cognitive impairment. Cochrane Database Syst Rev.

[10] Schneider LS (2011). Treatment with cholinesterase inhibitors and memantine of patients in the Alzheimer's disease neuroimaging initiative. Arch Neurol.

[11] Simons DJ (2016). Do “brain-training” programs work?. Psychol Sci Public Interest.

[12] Karlene Ball P, Berch DB, Helmers KF, Jobe JB, Mary, et al. Effects of cognitive training interventions with older adults: a randomized controlled trial. JAMA Neurol. 2002;288(18):2271–81. 10.1001/jama.288.18.2271 PMC2916176 12425704

[13] Belleville S (2006). Improvement of episodic memory in persons with mild cognitive impairment and healthy older adults: evidence from a cognitive intervention program. Dement Geriatr Cogn Disord.

[14] Suzuki H (2014). Cognitive intervention through a training program for picture book reading in community-dwelling older adults: a randomized controlled trial. BMC Geriatr.

[15] Bin-Yin Li H-DT, Qiao Y, Chen S-D. Mental training for cognitive improvement in elderly people: what have we learned from clinical and neurophysiologic studies? Curr Alzheimer Res. 2015;12(6):543–52. 10.2174/156720501206150716112918 26238812

[16] Jobe JB (2001). ACTIVE: a cognitive intervention trial to promote independence in older adults. Control Clin Trials.

[17] Barban F (2016). Protecting cognition from aging and Alzheimer's disease: a computerized cognitive training combined with reminiscence therapy. Int J Geriatr Psychiatry.

[18] Hill NT (2017). Computerized cognitive training in older adults with mild cognitive impairment or dementia: a systematic review and meta-analysis. Am J Psychiatry.

[19] Zhuang JP (2013). The impact of human-computer interaction-based comprehensive training on the cognitive functions of cognitive impairment elderly individuals in a nursing home. J Alzheimers Dis.

[20] Alberta MS, Steven T, DeKosky, Dickson D (2011). *The diagnosis of mild cognitive impairment due to Alzheimer’s disease: Recommendations from the National Institute on Aging-Alzheimer’s Association workgroups on diagnostic guidelines for Alzheimer’s disease*. Alzheimers Dement.

[21] Zhang ZX (2016). Rivastigmine patch in Chinese patients with probable Alzheimer's disease: a 24-week, randomized, double-blind parallel-group study comparing Rivastigmine patch (9.5 mg/24 h) with capsule (6 mg twice daily). CNS Neurosci Ther.

[22] Zhao Q (2012). Short-term delayed recall of auditory verbal learning test is equivalent to long-term delayed recall for identifying amnestic mild cognitive impairment. PLoS One.

[23] Zhao Q (2013). The Shape Trail test: application of a new variant of the trail making test. PLoS One.

[24] Zhao Q (2015). Auditory verbal learning test is superior to Rey-Osterrieth complex figure memory for predicting mild cognitive impairment to Alzheimer's disease. Curr Alzheimer Res.

[25] Scheltens P (1992). Atrophy of medial temporal lobes on MRI in “probable” Alzheimer's disease and normal ageing: diagnostic value and neuropsychological correlates. J Neurol Neurosurg Psychiatry.

[26] Zou QH (2008). An improved approach to detection of amplitude of low-frequency fluctuation (ALFF) for resting-state fMRI: fractional ALFF. J Neurosci Methods.

[27] Liu Y (2008). Regional homogeneity, functional connectivity and imaging markers of Alzheimer's disease: a review of resting-state fMRI studies. Neuropsychologia.

[28] Ding D (2016). Progression and predictors of mild cognitive impairment in Chinese elderly: a prospective follow-up in the shanghai aging study. Alzheimers Dement (Amst).

[29] Scheltens P, van de Pol L (2012). Impact commentaries. Atrophy of medial temporal lobes on MRI in “probable” Alzheimer’s disease and normal ageing: diagnostic value and neuropsychological correlates. J Neurol Neurosurg Psychiatry.

