# Supplementary material for: Shanghai cognitive intervention of mild cognitive impairment for delaying progress with longitudinal evaluation-a prospective, randomized controlled study (SIMPLE): rationale, design, and methodology
Source: BMC Neurol. 2018 Jul 24;18:103. doi: 10.1186/s12883-018-1100-x (PMC6057042; doi:10.1186/s12883-018-1100-x)
Supplement: Supplementary file 1 — Description of neuropsychological battery. Here is the description of neuropsychological assessments used in in the SIMPLE study, including their versions, scores and clinical significance. (DOCX 35 kb) [file 12883_2018_1100_MOESM1_ESM.docx]

Description of neuropsychological battery

Mini-Mental State Examination (MMSE) will be used to assess the global cognition function of MCI patients. It takes about 5 to 10 minutes to finish the 30-point questions which evaluates cognitive functions including orientation, memory, attention, calculation, language and visual-spatial functions[[1](#_ENREF_1)]. The culturally adapted Chinese version of the MMSE will be performed in screen and follow-up[[2](#_ENREF_2), [3](#_ENREF_3)]. The inclusion criterion of MMSE ranges between 24 and 28.

The Chinese version of the Alzheimer's Disease Assessment Scale-Cognitive Subscale (ADAS-cog) is regularly used to assess global cognitive dysfunction in AD clinical trials[[4-6](#_ENREF_4)]. ADAS-cog provides maximum information of all cognitive domains. The test estimates patients’ abilities of memory, naming, executive function, language, configuration, orientation and attention. A higher score means a greater cognition dysfunction. The scale score ranges from 0 to 70, with increasing scores indicating higher severity of global cognitive impairment. ADAS-Cog helps differentiate the cognitive impaired individuals from normal population, as well as contributes to evaluate the extent of cognitive impairment[[7](#_ENREF_7)].

Memory will be assessed using the Rey Auditory Verbal Learning Test-Huashan (Chinese) version (AVLT-H)[[8](#_ENREF_8)]. The patients will be required to memorize 12 unrelated words, with 20-minute interruption by other tests between memory encoding and recall. AVLT is stable of diagnoses and of predicting value of MCI progression. In the Chinese clinical setting, Zhao Q and Guo Q et al. suggested that the "one test" criterion AVLT is optimal in balancing sensitivity and specificity[[9](#_ENREF_9)].

Attention and executive function will be evaluated by Chinese modified version of Trail Making Test (TMT) [[10](#_ENREF_10)] and the Stroop Color-Word Test[[11](#_ENREF_11)]. TMT evaluates functions of rapid visual search, visual spatial ordering and mental flexibility. The difference between time consumed in TMT-B and TMT-A represents the impairment of executive functions. TMT-B is reported to be significantly predictive for the development of dementia in amnestic MCI[[12](#_ENREF_12)]. In the Stroop test, patients will be required to read words of colors (the first trial) , then to read colors (the second trial) and finally name the colors of words (the third trial). The ratio of time consumed in the third trial and the first trial indicates attention/executive function.

Language will be assessed by a modified Chinese-version Boston naming test (30-item)[[13](#_ENREF_13)]. This test requires patients to name 30 pictures with certain sequence. This version had demonstrated good validity for detecting naming impairment in Chinese-speaking patients in Shanghai[[14](#_ENREF_14)].Visual spatial ability and memory will be evaluated by the Rey-Osterrieth complex figure test (CFT). Patients will be required to copy a complicated line drawing and to recall the picture and draw it[[14](#_ENREF_14)].

Activities of daily living (ADL)will be assessed by authorized Chinese version of Alzheimer's Disease Cooperative Study-Activities of Daily Living (ADCS-ADL)[[6](#_ENREF_6)]. Patients’ caregivers will be required to recall and answer 23 questions about ADL of patients in the last 4 weeks. Two major types of living activities involved are basic ADL like eating and dressing, and instrumental ADL like talking and shopping. The existence of depression will be found out by Hamilton Depression Scale (HAMD-17 items)[[15](#_ENREF_15)], which are [self-report](https://en.wikipedia.org/wiki/Self-report_inventory) [assessment](https://en.wikipedia.org/wiki/Psychological_assessment)s with questions about mood and interest in daily activities. The score higher than 7 points indicates existence of depression, and a higher score indicates more severe depression.

1. Tombaugh, T.N. and N.J. McIntyre, *The mini-mental state examination: a comprehensive review.* J Am Geriatr Soc, 1992. **40**(9): p. 922-35.

2. Yang, Z., et al., *Optimal Cutoff Scores for Alzheimer's Disease Using the Chinese Version of Mini-Mental State Examination Among Chinese Population Living in Rural Areas.* Am J Alzheimers Dis Other Demen, 2016. **31**(8): p. 650-657.

3. Katzman, R., et al., *A Chinese version of the Mini-Mental State Examination; impact of illiteracy in a Shanghai dementia survey.* J Clin Epidemiol, 1988. **41**(10): p. 971-8.

4. Zhang, Z.X., et al., *Rivastigmine Patch in Chinese Patients with Probable Alzheimer's disease: A 24-week, Randomized, Double-Blind Parallel-Group Study Comparing Rivastigmine Patch (9.5 mg/24 h) with Capsule (6 mg Twice Daily).* CNS Neurosci Ther, 2016. **22**(6): p. 488-96.

5. Cheng, C.P., et al., *Would transcranial direct current stimulation (tDCS) enhance the effects of working memory training in older adults with mild neurocognitive disorder due to Alzheimer's disease: study protocol for a randomized controlled trial.* Trials, 2015. **16**: p. 479.

6. Tian, J., et al., *The efficacy and safety of Fufangdanshen tablets (Radix Salviae miltiorrhizae formula tablets) for mild to moderate vascular dementia: a study protocol for a randomized controlled trial.* Trials, 2016. **17**(1): p. 281.

7. Benge, J.F., et al., *How well do the ADAS-cog and its subscales measure cognitive dysfunction in Alzheimer's disease?* Dement Geriatr Cogn Disord, 2009. **28**(1): p. 63-9.

8. Guo, Q., et al., *A comparison study of mild cognitive impairment with 3 memory tests among Chinese individuals.* Alzheimer Dis Assoc Disord, 2009. **23**(3): p. 253-9.

9. Zhao, Q., et al., *Auditory Verbal Learning Test is Superior to Rey-Osterrieth Complex Figure Memory for Predicting Mild Cognitive Impairment to Alzheimer's Disease.* Curr Alzheimer Res, 2015. **12**(6): p. 520-6.

10. Zhao, Q., et al., *The Shape Trail Test: application of a new variant of the Trail making test.* PLoS One, 2013. **8**(2): p. e57333.

11. Zhou, B., et al., *Executive function predicts survival in Alzheimer disease: a study in Shanghai.* J Alzheimers Dis, 2010. **22**(2): p. 673-82.

12. Bondi, M.W., et al., *Cognitive and neuropathologic correlates of Stroop Color-Word Test performance in Alzheimer's disease.* Neuropsychology, 2002. **16**(3): p. 335-43.

13. Cheung, R.W., M.C. Cheung, and A.S. Chan, *Confrontation naming in Chinese patients with left, right or bilateral brain damage.* J Int Neuropsychol Soc, 2004. **10**(1): p. 46-53.

14. Ma, J., Y. Zhang, and Q. Guo, *Comparison of vascular cognitive impairment--no dementia by multiple classification methods.* Int J Neurosci, 2015. **125**(11): p. 823-30.

15. Hamilton, M., *A rating scale for depression.* J Neurol Neurosurg Psychiatry, 1960. **23**: p. 56-62.
